# Lifestyle Modification Strategies to Counteract the World Epidemic Growth of Obesity and Diabetes

**DOI:** 10.1155/2014/640409

**Published:** 2014-12-28

**Authors:** Pierpaolo De Feo, Jean-Michel Boris, Claudio Maffeis

**Affiliations:** ^1^Healthy Lifestyle Institute (CURIAMO), University of Perugia, Via G. Bambagioni 19, 06126 Perugia, Italy; ^2^EPODE European Network, Proteines, 11 rue Galvani, 75017 Paris, France; ^3^Regional Center for Pediatric Diabetes, Clinical Nutrition & Obesity, Department of Life & Reproduction Sciences, University of Verona, Via Bengasi 4, 37134 Verona, Italy

Obesity and type 2 diabetes are increasing all over the world at an alarming rate due to unhealthy lifestyles. The fight against childhood obesity represents one of the biggest social and health problems all over the world. Diabetes mellitus reduces quality of life and life expectancy and represents a serious economic burden. The correction of unhealthy lifestyles is possible through cost-effective measures and it might prevent about 80% of cases of heart disease, stroke, and type 2 diabetes and 40% of cancers. At present, there is a need to advance in the science of delivering health promotion and reproducible multidisciplinary models for the prevention and treatment of obesity and diabetes using lifestyle change. Particularly alarming is the childhood obesity which represents the most common metabolic disorder in children and adolescents. Three reasons other than the high prevalence justify the urgency of treating and preventing childhood obesity: (i) persistency of childhood obesity into adulthood; (ii) metabolic and nonmetabolic morbidity obesity associated; (iii) higher mortality in adulthood. The American Academy of Pediatrics published guidelines with the targets for prevention and treatment of childhood obesity, based on consistent scientific evidence. Nutrition and physical activity are the main tools of intervention. Recent evidence suggests that prevention should start early, from intrauterine life and infancy, when the sensitivity of the organism to metabolic long-term programming processes is high and the potential impact of the intervention may be considerable. The discouraging results of the impact of available interventions in prevention and treatment of childhood obesity stimulate new emphasis in both clinical and experimental research in the field.

The present special issue has been published to enlarge the knowledge in the field of the battle against childhood and adult obesity and type 2 diabetes.

Four papers address the issue of childhood obesity prevention and treatment. G. Valerio et al. report that perception of task's difficulty level may reflect an actual difficulty in obese children. These findings have practical implications for approaching physical activity in obese children. In order to design exercise programs that allow safe and successful participation it is relevant to explore both the perception of a task's difficulty level and physical performance of obese children. H. Zheng and coworkers originally looked for possible associations between the metabolome and gender, pubertal development, and physical activity in overweight adolescents, which is an important subject group to approach in the prevention of obesity and life-style related diseases. No relations between physical activity and the metabolome could be identified, whereas gender differences in the metabolome are being commenced already in childhood and might be used for identification of individuals susceptible to an early pubertal development. C. Mazzeschi et al. describe the design of the intervention programme EUROBIS (Epode Umbria Region Obesity Intervention Study), an EPODE community-based intervention combined with a clinical trial, which provides an innovative valuable model to address the childhood obesity prevention and treatment. Finally, A. Verrotti et al. explored a possible relationship between prevalence, frequency, and severity of migraine and obesity in children. Interestingly, data from literature reviewed in this paper suggest that obesity can be linked with migraine prevalence, frequency, and disability in both paediatric and adult subjects, suggesting the importance for the quality of life of children of lifestyle interventions.

Regarding adult obesity, the paper by K. M. J. Azar et al. examined whether baseline BMI may influence behavioural weight-loss treatment effectiveness. The authors show that participants with baseline BMI had greater reductions in mean BMI, body weight, and waist circumference in the coach-led group intervention, compared to usual care and the self-directed individual intervention. These results indicate the importance of taking into account individual's baseline BMI when developing or recommending lifestyle focused treatment strategy. B. Strasser and D. Pesta reviewed the evidence about the role of resistance training in counteracting the metabolic dysfunction in patients with type 2 diabetes. The authors report several beneficial adaptations exerted by resistance training which include increased GLUT4 translocation in skeletal muscle, increased insulin sensitivity, increased energy expenditure, and excess postexercise oxygen consumption. This paper supports the importance of using combined aerobic and resistance exercise as a therapeutic tool for type 2 diabetic patients. A positive message about the beneficial effects of walking in obesity and type 2 diabetes comes out of the paper of C. Mazzeschi et al. who originally report the effects of prolonged exercise (about 400 km walk over 2 weeks) on several health and psychological outcomes. The results of this study demonstrate that long-distance treks are a safe activity for trained overweight/obese people that should be recommended because they improve mood, health status, and the relationship of participants with themselves and with the regular practice of exercise with effects similar to those obtained by healthy normal weight subjects.

*Pierpaolo De Feo* *Jean-Michel Boris* *Claudio Maffeis*

